# Supplementary material for: Enhancing perceptual, attentional, and working memory demands through variable practice schedules: insights from high-density EEG multi-scale analyses
Source: Cereb Cortex. 2024 Nov 6;34(11):bhae425. doi: 10.1093/cercor/bhae425 (PMC11538921; doi:10.1093/cercor/bhae425)
Supplement: Supplementary_material_bhae425 [file supplementary_material_bhae425.docx]

**SUPPLEMENTARY MATERIAL**


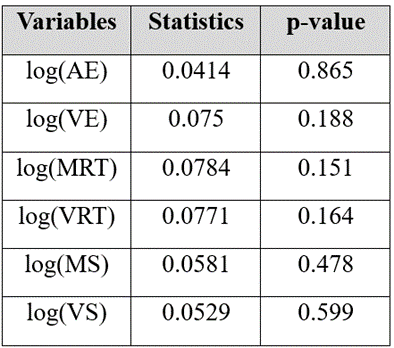


Table S1. Result of the Kolmogorov-Smirnov normality test for each of the 6 log-transformed behavioral variables. *P*-value above 0.05 indicates the non-violation of the normality hypothesis.


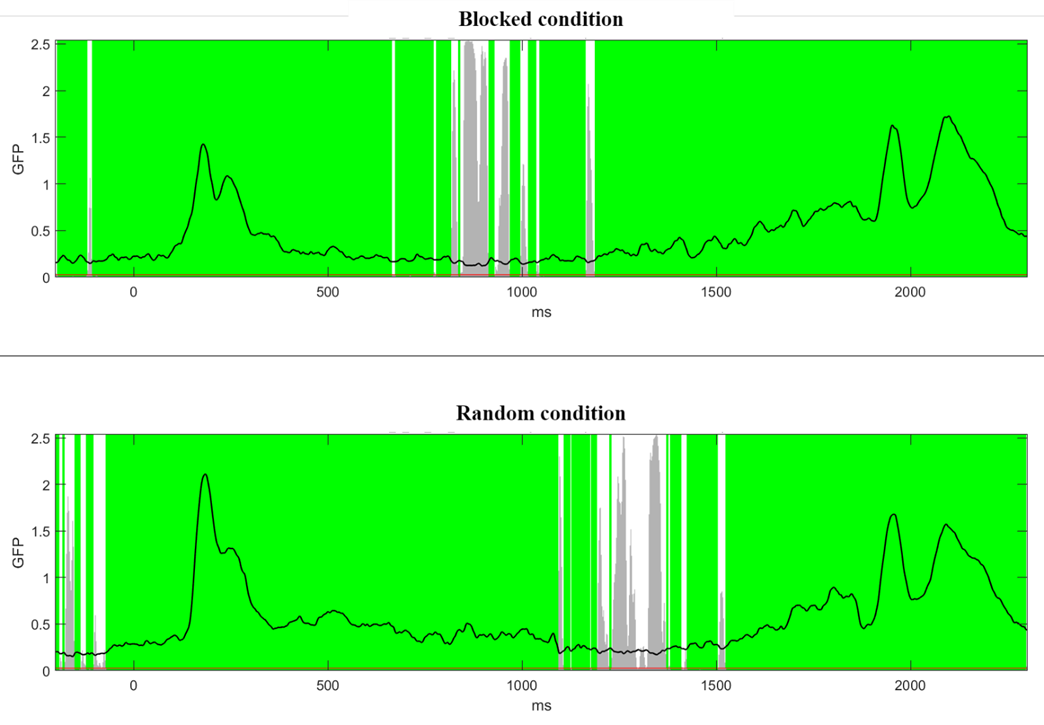


Figure S1. Result of Topographical Consistency Test for each condition. The figure depicts the GFP dynamic over the epoch. The green areas represent period of consistent topographies within each condition. Grey areas represents period of inconsistent topographies within each condition.


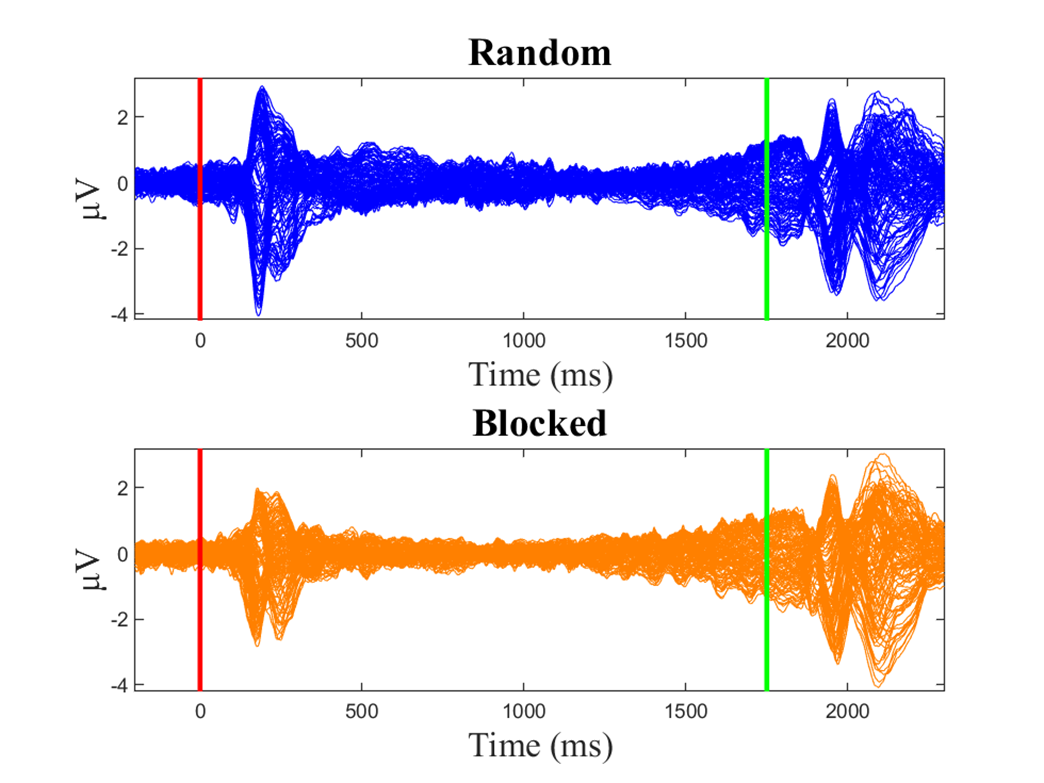


Figure S2. Mean ERPs of all electrodes for random (blue) and blocked (orange) conditions. The red line marks the moment when the distance to be executed is displayed and the green line signifies the imperative stimulus when participants are required to aim at the target.
